# Clinical characteristics and course of pulmonary artery stump thrombosis following lung cancer surgery: A retrospective study from tertiary care hospital

**DOI:** 10.1097/MD.0000000000036879

**Published:** 2024-01-12

**Authors:** Ji-Eun Park, Seung-Ick Cha, Deok Heon Lee, Eung Bae Lee, Sun Ha Choi, Yong Hoon Lee, Hyewon Seo, Seung-Soo Yoo, Shin-Yup Lee, Jaehee Lee, Chang-Ho Kim, Jae-Yong Park

**Affiliations:** a Department of Internal Medicine, School of Medicine, Kyungpook National University, Daegu, South Korea; b Department of Thoracic and Cardiovascular Surgery, School of Medicine, Kyungpook National University, Daegu, South Korea.

**Keywords:** lung cancer, prognosis, pulmonary embolism, surgery, thrombosis

## Abstract

The data regarding pulmonary artery stump thrombosis (PAST) after lung cancer surgery are insufficient. The aim of the present study was to evaluate the incidence, clinical characteristics, and prognosis of PAST. We retrospectively investigated the incidence and clinical characteristics of PAST among patients who underwent lung resection for lung cancer at 2 institutions. We compared the clinical parameters between PAST and pulmonary embolism (PE) and examined the clinical course of patients with PAST. Of the 1885 patients, PAST was found in 36 patients (1.9%). Right lower lobectomy (n = 13) and middle-lower bilobectomy (n = 9) were the most common types of surgery. The median time interval from lung resection to the detection of PAST was 3.8 months. Immobilization and a history of cerebrovascular disease were not observed in the PAST group. Most of the patients with PAST (91.7%) were diagnosed incidentally, whereas many patients with PE (75.9%) were symptomatic at the time of diagnosis. During the follow-up, one patient (2.8%) had contralateral PE complications. However, no patients in the PAST group experienced pulmonary thromboembolism-related in-hospital death or adverse outcomes. There was no difference in the prognosis of patients with PAST according to the administration of anticoagulation. PAST was rarely detected in lung cancer patients on follow-up chest computed tomography after lung resection. Patients with PAST were asymptomatic in most cases and had relatively favorable clinical outcomes. However, these patients are at risk of contralateral PE, despite its rarity.

## 1. Introduction

Patients with lung cancer undergoing surgical treatment are at a high risk of developing venous thromboembolism (VTE) with an incidence of 5% to 20%.^[1–3]^ According to Virchow triad,^[4]^ hypercoagulability due to lung cancer, endothelial injury, and blood stasis after major lung surgery may contribute to the development of thromboembolism in this population.^[2,5]^ However, pulmonary artery stump thrombosis (PAST) is another complication after lung cancer surgery, and it is currently considered as a disease entity that is different from pulmonary embolism (PE).^[6]^ PAST was first reported by Crafoord in 1938,^[7]^ and Chunag et al reported 2 autopsy findings of vascular stump thrombosis after pneumonectomy with contralateral PE in 1966.^[8]^ Thereafter, several case reports and studies regarding the incidence, risk factors, and prognosis of PAST were published.^[6,9,10]^ The incidence of PAST has been reported to vary according to the degree of pulmonary resection, and stump length was related to the development of PAST.^[11–14]^ Although the optimal treatment of PAST is still unknown, the prognosis of patients with PAST is assumed to be good.

The clinical importance of PAST will continue to increase as the number of patients undergoing surgical treatment for lung cancer increases and postoperative computed tomography (CT) image-based surveillance strategies improve.^[15,16]^ It is also important to distinguish PAST from PE because the natural history, treatment, and prognosis of these 2 distinct diseases may differ. However, data regarding PAST are insufficient due to the limited number of studies. Therefore, the aim of this study was to evaluate the incidence, clinical characteristics, and prognosis of patients with PAST and compare the clinical characteristics of PAST and PE.

## 2. Methods

### 2.1. Study population and design

**Figure 1.**
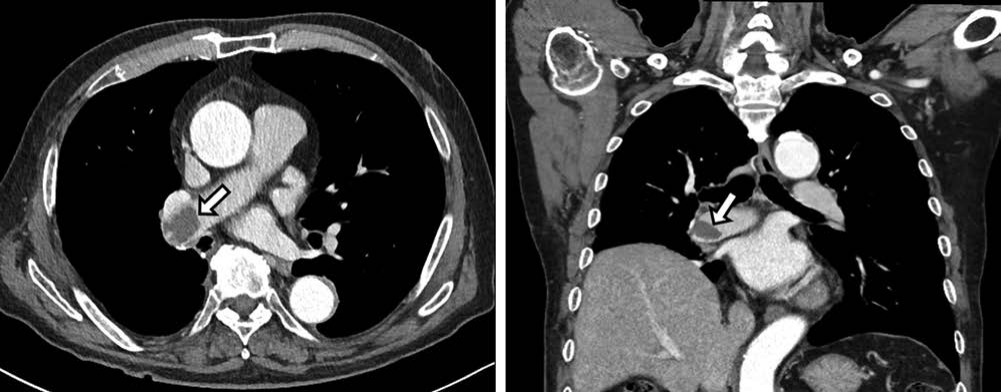
Chest computed tomographic images of pulmonary artery stump thrombosis. A filling defect (arrow) is observed in the right pulmonary artery after a right lower lobectomy.

This study was conducted at 2 tertiary referral hospitals in South Korea, Kyungpook National University Hospital (KNUH) and Kyungpook National University Chilgok Hospital (KNUCH). We retrospectively collected the data of all consecutive patients who underwent lung resection for lung cancer at KNUH between 2008 and 2019, and at KNUCH between 2011 and 2019. Of these patients, those who underwent lobectomy, bilobectomy, or pneumonectomy and those who had follow-up enhanced chest CT scans within 1 year after surgery were included. Patients who underwent sublobar resection, those with deep vein thrombosis (DVT), and those without medical records or follow-up chest CT scans were excluded. PAST was diagnosed by the presence of a solitary filling defect with soft tissue attenuation confined within the pulmonary arterial stump (Fig. 1).

To compare PAST with PE, patients who were diagnosed with PE at KNUH between 2009 and 2019 were allocated to the control group. Of these, only patients with pulmonary emboli in the lobar or more proximal pulmonary arteries were included. The study protocol was approved by each Institutional Review Board of KNUH (KNUH 2022-08-034) and KNUCH (KNUCH 2022-11-002), which waived the requirement for written informed consent because of the retrospective nature of the study.

### 2.2. Data acquisition

Data regarding lung cancer, operative procedures, and the location of stump thrombi were collected from the medical records of patients with PAST. Clinical characteristics, comorbidities, and anticoagulant treatment were also obtained. PE severity index (PESI) and simplified PESI (sPESI) scores were retrospectively calculated for patients with PAST and PE.^[17]^ We also evaluated the presence of right ventricular (RV) dilation as an independent predictor of adverse outcomes.^[18]^ RV dilation was defined when the right-to-left ventricular diameter ratio was ≥ 1. The diameter of each ventricle was measured by the maximal distance between the ventricular endocardium and the interventricular septum, perpendicular to the long axis of the heart, in the standard axis view. Adverse outcomes were defined as pulmonary thromboembolism (PTE)-related in-hospital mortality and serious conditions, including cardiac arrest, the need for vasopressors, and impending respiratory failure. The onset of PAST was determined as the date of chest CT examination when the stump thrombus was first detected. The clinical course of PAST was classified as follows: complete resolution when the thrombus completely disappeared, partial resolution when the thrombus size decreased but remained, stabilization when the thrombus size was unchanged, and progression when the thrombus size increased.

### 2.3. Statistical analysis

Statistical analyses were performed using IBM SPSS Statistics for Windows, version 22.0 (IBM Corp., Armonk, New York, USA). Continuous variables are expressed as the mean with standard deviation (SD) or median with interquartile range (IQR), and differences between the groups were analyzed using the Student *t* test or Fisher exact test. Categorical variables are expressed as absolute values and percentages and analyzed using the χ^2^ test. Variables with *p*-values of <0.05 were considered statistically significant.

## 3. Results

### 3.1. Incidence and clinical characteristics of patients with pulmonary artery stump thrombosis

**Figure 2.**
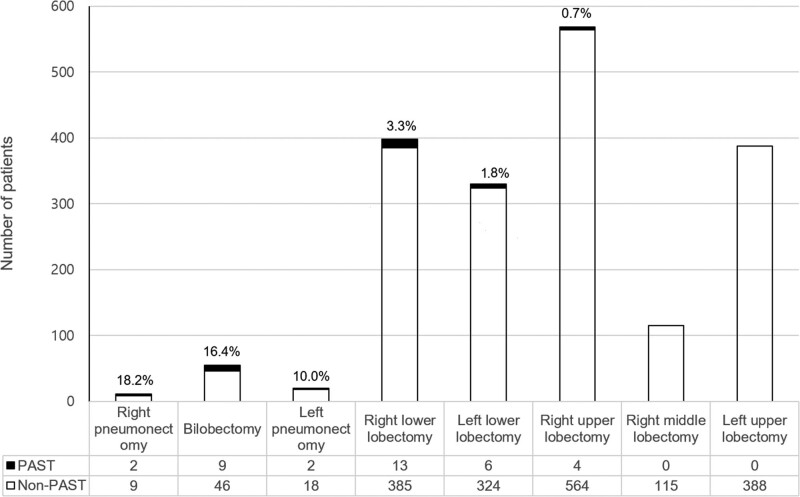
Distribution of pulmonary artery stump thrombosis according to the type of lung resection. PAST = pulmonary artery stump thrombosis.

PAST was found in 36 (1.9%) of the 1885 patients who fulfilled the inclusion criteria. The clinical characteristics of patients with PAST are summarized in Table 1. Lung adenocarcinoma and squamous cell carcinoma were diagnosed in 17 patients (47.2%) and 14 patients (38.9%), respectively. Stage I lung cancer was diagnosed in 17 patients (47.2%), followed by stage II (n = 10) and stage III (n = 8), according to the 8 edition of lung cancer stage classification,^[19]^ and one-third of the patients with PAST received adjuvant chemotherapy or radiotherapy. Right lower lobectomy (n = 13) and middle-lower bilobectomy (n = 9) were the most common types of surgery, followed by left lower lobectomy (n = 6), right upper lobectomy (n = 4), right pneumonectomy (n = 2), and left pneumonectomy (n = 2). Among all patients who underwent lung cancer surgery during the study period, the highest incidence of PAST occurred after right pneumonectomy (18.2%), followed by bilobectomy (16.4%), and left pneumonectomy (10.0%) (Fig. 2). The median time interval from lung resection to the first detection of PAST was 3.8 months (IQR, 2.7–8.6 months).

**Table 1:** Clinical characteristics of pulmonary artery stump thrombosis (n = 36).

Histologic type of lung cancer	
Adenocarcinoma	17 (47.2)
Squamous cell carcinoma	14 (38.9)
Others*	5 (13.9)
Pathologic stage	
I	17 (47.2)
II	10 (27.8)
III	8 (22.2)
IV	1 (2.8)
Adjuvant therapy	
Chemotherapy	4 (11.1)
Radiotherapy	2 (5.6)
Chemoradiotherapy	5 (13.9)
Operative approach	
VATS	13 (36.1)
Thoracotomy	23 (63.9)
Lymph node dissection	
MLND	26 (72.2)
MLS	8 (22.2)
Operation time, min	195 (160–263)
Type of surgical resection	
Right lower lobe lobectomy	13 (36.1)
Bilobectomy	9 (25.0)
Left lower lobe lobectomy	6 (16.7)
Right upper lobe lobectomy	4 (11.1)
Right pneumonectomy	2 (5.6)
Left pneumonectomy	2 (5.6)
Location of PAST	
Right pulmonary artery	12 (33.3)
Right lower lobar artery	11 (30.6)
Left pulmonary artery	6 (16.7)
Right interlobar artery	4 (11.1)
Left lower lobar artery	2 (5.6)
Truncus anterior	1 (2.8)
Left upper lobar artery	0 (0)
First detection of PAST after lung resection, months	3.8 (2.7–8.6)

Data are expressed as median (interquartile range) or n (%).

MLND = mediastinal lymph node dissection, MLS = mediastinal lymph node sampling, PAST = pulmonary artery stump thrombosis, VATS = video-assisted thoracoscopic surgery.

* Others include small cell carcinoma, large cell carcinoma, adenosquamous carcinoma, sarcomatoid carcinoma, and combined carcinoma (squamous cell carcinoma and large cell carcinoma).

### 3.2. Comparison of clinical characteristics between patients with pulmonary artery stump thrombosis and pulmonary embolism

The clinical characteristics of the patients with PAST were compared to those of PE patients (n = 790) (Table 2). Although the mean age was similar between the 2 groups, there were significantly more male patients (30 [83.3%] vs 302 [38.2%], *P* < .001) and ever-smokers (29 [80.6%] vs 263 [33.3%], *P* < .001) in the PAST group than in the PE group. Regarding comorbidities, the percentage of patients with chronic pulmonary disease was significantly higher in the PAST group (8 [22.2%] vs 79 [10%], *P* = .044). Different from the PE group, immobilization, one of the major transient risk factors of VTE, was not observed in the PAST group. A history of cerebrovascular disease was not found in patients with PAST. Most of the patients with PAST (91.7%) were diagnosed incidentally, whereas many patients with PE (75.9%) presented with symptomatic PEs at the time of diagnosis.

**Table 2:** Comparison of clinical characteristics between PAST and PE groups.

Characteristics	PAST (n = 36)	PE (n = 790)	*P* value
Age, yr	70.1 ± 6.6	68.6 ± 13.9	.200
Male sex	30 (83.3)	302 (38.2)	<.001
BMI, kg/m^2^	23.5 ± 3.0	23.8 ± 3.8	.623
Smoking			
Ever-smoker	29 (80.6)	263 (33.3)	<.001
Pack-years	48.7 ± 24.4	24.2 ± 22.5	<.001
Comorbidities			
Malignancy	36 (100)	135 (17.1)	<.001
Hypertension	11 (30.6)	243 (30.8)	.979
Chronic pulmonary disease*	8 (22.2)	79 (10)	.044
Atrial fibrillation	4 (11.1)	34 (4.3)	.078
Diabetes mellitus	3 (8.3)	114 (14.4)	.305
Immobilization	0 (0)	362 (45.8)	<.001
Cerebrovascular disease	0 (0)	95 (12.0)	.027
Previous history of VTE	0 (0)	73 (9.2)	.066
Congestive heart failure	0 (0)	31 (3.9)	.393
Obesity	0 (0)	7 (0.9)	>.999
PTE-related symptoms			
Any symptom	3 (8.3)	600 (75.9)	<.001
Incidental CT finding	33 (91.7)	190 (24.1)	<.001
Dyspnea	3 (8.3)	382 (48.4)	<.001
Chest pain	0 (0)	97 (12.3)	.016
Leg swelling or pain	0 (0)	79 (10.0)	.041
Syncope or dizziness	0 (0)	29 (3.7)	.632
Hemoptysis	0 (0)	28 (3.5)	.629
PESI score	112.5 ± 12.9	90.1 ± 27.5	<.001
PESI class IV-V	27 (75.0)	191 (24.2)	<.001
Simplified PESI, high risk	36 (100.0)	458 (58.0)	<.001
PTE-related in-hospital death	0 (0)	13 (1.6)	>.999
Adverse outcomes†	0 (0)	70 (8.9)	.065
Anticoagulation	15 (41.7)	775 (98.1)	<.001
CT findings			
RV dilation	5 (13.9)	362 (45.9)	<.001
Largest PA site involved by PTE			.219
Right or left PA or more proximal	19 (52.8)	335 (42.4)	
Interlobar or lobar	17 (47.2)	455 (57.6)	

Data are expressed as mean ± standard deviation or n (%).

BMI = body mass index, CT = computed tomography, PA = pulmonary artery, PAST = pulmonary artery stump thrombosis, PE = pulmonary embolism, PESI = pulmonary embolism severity index, PTE = pulmonary thromboembolism, RV = right ventricle, VTE = venous thromboembolism.

* Chronic pulmonary diseases include chronic obstructive pulmonary disease, asthma, interstitial lung diseases, tuberculosis-destroyed lung, bronchiectasis, and bronchial anthracofibrosis.

† Adverse outcomes include cardiac arrest or the need for vasopressors, inotropes, or impending respiratory failure.

The proportion of PESI class IV to V (27 [75.0%] vs 191 [24.2%], *P* < .001) and high risk sPESI (36 [100.0%] vs 458 [58.0%], *P* < .001) was significantly higher in the PAST group compared to the PE group. RV dilation was found less often in the PAST group than in the PE group (5 [13.9%] vs 362 [45.9%], *P* < .001). For the treatment of thromboembolism, less than half of the PAST group started anticoagulants, whereas most of the patients with PE received anticoagulation (15 [41.7%] vs775 [98.1%], *P* < .001). Of the 15 patients who received anticoagulants in the PAST group, direct oral anticoagulants were most commonly prescribed (n = 8), followed by warfarin (n = 5) and low molecular weight heparin (n = 2). No patient in the PAST group experienced PTE-related in-hospital death or adverse outcome.

### 3.3. Clinical course of pulmonary artery stump thrombosis and effect of anticoagulation

Thirty-four patients with PAST (94.4%) had follow-up chest CTs and were evaluated for changes in stump thrombi. The median time between the first CT follow-up and the diagnosis of PAST was 2.9 months (IQR, 1.8–4.3 months) (Table 3). Complete resolution was found in 15 patients (44.1%) in the first chest CT follow-up. Partial resolution and stabilization were found in 9 patients (26.5%) and 10 patients (29.4%), respectively, and none of the patients experienced progression. The final chest CT scan of each patient showed complete resolution in 22 patients (64.7%), partial resolution in 6 patients (17.6%), stabilization in 4 patients (11.8%), and progression in 2 patients (5.9%). In 22 patients with complete resolution, thrombi disappeared after a median duration of 4.6 months (IQR, 2.7–7.8 months), and the median follow-up time without recurrence was 16.7 months (IQR, 1.2–36.4 months). During the follow-up, contralateral PE occurred in one patient (2.8%). The patient did not receive anticoagulation after the diagnosis of left-sided PAST, and right PE developed 2 years after PAST.

**Table 3:** Clinical course of patients with PAST (n = 34).

Time to first CT follow-up after PAST, months	2.9 (1.8–4.3)
Findings at first CT follow-up	
Complete resolution	15 (44.1)
Partial resolution	9 (26.5)
Stabilization	10 (29.4)
Progression	0 (0)
Findings at last CT follow-up	
Complete resolution	22 (64.7)
Partial resolution	6 (17.6)
Stabilization	4 (11.8)
Progression	2 (5.9)
Time to complete resolution, months (n = 22)	4.6 (2.7–7.8)
Duration of follow-up without recurrence, months (n = 22)	16.7 (1.2–36.4)
Occurrence of contralateral pulmonary embolism	1 (2.8)

Data are expressed as median (interquartile range) or n (%).

CT = computed tomography, PAST = pulmonary artery stump thrombosis.

PAST prognosis was compared between 13 patients who received anticoagulants and 21 patients who did not receive anticoagulants. Stump thrombosis recurred in 2 anticoagulated patients (15.4%), although they were still receiving anticoagulants after the first complete disappearance of stump thrombi. Two patients (9.5%) who did not receive anticoagulation experienced thrombi progression. There was no difference in the prognosis of stump thrombosis according to the administration of anticoagulation.

## 4. Discussion

In the present study, we investigated the incidence, clinical characteristics, and prognosis of patients with PAST diagnosed based on follow-up chest CT after lung cancer surgery. The overall incidence of PAST after lung resection for lung cancer was 1.9%, and more than three-quarters of PAST occurred in the right pulmonary arteries. Most PAST was diagnosed incidentally, and several different clinical characteristics were identified compared to patients with PE. Although fewer than half of the PAST patients received anticoagulation, the prognosis of PAST was favorable.

PAST was diagnosed at a median of 3.8 months after lung resection. Although the delayed presentation of PAST has been reported in several cases (late PAST),^[20–22]^ most PAST cases occur within 12 months after the surgery (early PAST).^[9]^ Previous studies reported that the incidence of PAST after lobectomy or pneumonectomy was 2 to 5%,^[13,14]^ consistent with our results. However, the incidence of PAST increased to 12% in patients undergoing pneumonectomy.^[11,12]^ This difference is probably because prolonged vascular manipulations during more complex surgery may increase the risk of endothelial injury, thereby increasing the chance of thrombus formation at the stump site.^[6]^ In addition to the extent of surgery, the ligation technique, which causes more damage to the intimal surface of the pulmonary artery, was also associated with the development of PAST.^[14,23]^ In the present study, PAST occurred more frequently after a right-sided lobectomy (2.4%) than after a left-sided one (1.1%), albeit statistical significance was not evaluated because of the small sample size. Previous studies reported that the right stump had a greater risk of PAST than the left,^[11,13]^ but other studies have also shown nearly equal rates on both sides,^[12,14]^ leaving the issue controversial. However, most of the studies consistently reported that a longer vascular stump was significantly related to the development of PAST, regardless of the stump site.^[11,12,14]^ A longer vascular stump on the right side may cause more blood turbulence or stasis leading to thrombus formation. However, the causal relationship between the stump site and the occurrence of PAST needs to be further evaluated.

Noteworthy, PAST has several distinctive clinical characteristics compared to PE. First, patients with PAST did not have well-known risk factors for VTE, such as immobilization or a previous history of VTE.^[24]^ Second, most of the patients with PAST were diagnosed incidentally on their follow-up CT, whereas more than 75% of the patients with PE experienced symptoms. Moon *et al* reported that all of the patients with vascular stump thrombosis, including both arterial and venous stumps, were asymptomatic.^[6]^ Therefore, these findings suggest that PAST has a different pathophysiology from PE, and thus, the treatment indication and modality might be different. Lastly, in terms of clinical outcome, 75% of the patients in the PAST group were included in the high-risk group according to PESI scores. However, none of the patients experienced PTE-related in-hospital death or adverse outcomes in the PAST group. The percentage of RV dilation was significantly lower in the PAST group than in the PE group, suggesting a benign outcome of PAST. Therefore, the PESI score may not be appropriate for predicting PAST prognosis, and further studies are needed to find the prognostic factors in these patients.

Although there was no difference in outcomes regardless of the treatment in the present study, the need for anticoagulation is still controversial. Anticoagulation therapy has been suggested for the following indications: late PAST, convex-shaped or floating thrombus, or declining pulmonary status.^[12,13,21]^ The possible complications of PAST include an extension of in situ thrombi, embolization to the contralateral lung, or chronic pulmonary hypertension.^[20,21,25]^ In our study, one patient experienced PE in the contralateral lung. No fatal outcomes occurred in these patients, but contralateral lung embolization can be lethal. Therefore, although PAST is usually diagnosed incidentally and the prognosis seems to be favorable, anticoagulation should be considered in patients with an increased risk of complications.

Several limitations should be noted in the current study. First, PAST was diagnosed clinically rather than confirmed by pathologic findings. However, in real clinical practice, the diagnosis of PAST is usually made after excluding other causes of filling defects in the pulmonary artery, such as PE or tumor recurrence in the vascular stump.^[11]^ In the current study, patients with DVT were excluded to rule out the possibility of embolism. There are no reliable radiologic findings that can clearly differentiate in situ pulmonary thrombosis from PE, and this is also difficult in an autopsy.^[26]^ Tumor recurrence could be ruled out because most of the patients (94.4%) were able to undergo follow-up chest CT scans. Second, the timing of chest CT follow-up differed between the patients. Therefore, the exact time of thrombus development and disappearance could not be determined. Third, selection bias was inevitable due to its retrospective study design, and the statistical power of the analysis was weak due to the small number of patients with PAST.

In conclusion, PAST was detected in 1.9% of lung cancer patients on follow-up chest CT after lung resection. Unlike PE patients, most PAST patients were asymptomatic and had relatively favorable clinical outcomes. However, contralateral PE in the remaining lung following PAST can be fatal. Therefore, future studies should establish individualized guidelines for anticoagulation in patients with PAST.

## Acknowledgments

We would like to thank Harrisco (en.harrisco.net) for English language editing.

## Author contributions

**Conceptualization:** Ji-Eun Park, Seung-Ick Cha.

**Data curation:** Ji-Eun Park, Seung-Ick Cha.

**Formal analysis:** Ji-Eun Park, Seung-Ick Cha, Deok Heon Lee, Eung Bae Lee.

**Investigation:** Yong Hoon Lee, Jaehee Lee, Chang-Ho Kim.

**Methodology:** Sun Ha Choi, Hyewon Seo, Seung-Soo Yoo, Shin-Yup Lee, Jae-Yong Park.

**Supervision:** Ji-Eun Park, Seung-Ick Cha.

**Writing – original draft:** Ji-Eun Park, Seung-Ick Cha.

**Writing – review & editing:** Deok Heon Lee, Eung Bae Lee, Sun Ha Choi, Yong Hoon Lee, Hyewon Seo, Seung-Soo Yoo, Shin-Yup Lee, Jaehee Lee, Chang-Ho Kim, Jae-Yong Park.
